# Augmenting aesthetic chills using a wearable prosthesis improves their downstream effects on reward and social cognition

**DOI:** 10.1038/s41598-020-77951-w

**Published:** 2020-12-10

**Authors:** A. J. H. Haar, A. Jain, F. Schoeller, P. Maes

**Affiliations:** 1 Fluid Interfaces Group, Media Lab, Massachusetts Institute of Technology, Cambridge, USA; 2 Centre de Recherches Interdisciplinaires, Paris, France

**Keywords:** Emotion, Reward, Somatosensory system, Human behaviour

## Abstract

Previous studies on aesthetic chills (i.e., psychogenic shivers) demonstrate their positive effects on stress, pleasure, and social cognition. We tested whether we could artificially enhance this emotion and its downstream effects by intervening on its somatic markers using wearable technology. We built a device generating cold and vibrotactile sensations down the spine of subjects in temporal conjunction with a chill-eliciting audiovisual stimulus, enhancing the somatosensation of cold underlying aesthetic chills. Results suggest that participants wearing the device experienced significantly more chills, and chills of greater intensity. Further, these subjects reported sharing the feelings expressed in the stimulus to a greater degree, and felt more pleasure during the experience. These preliminary results demonstrate that emotion prosthetics and somatosensory interfaces offer new possibilities of modulating human emotions from the bottom-up (body to mind). Future challenges will include testing the device on a larger sample and diversifying the type of stimuli to account for negatively valenced chills and intercultural differences. Interoceptive technologies offer a new paradigm for affective neuroscience, allowing controlled intervention on conscious feelings and their downstream effects on higher-order cognition.

## Introduction

Body perception plays a critical role in making emotional signals accessible to consciousness through feelings and their associated embodied effects^1–3^. Internal representations of the external world are actively mediated by physical feelings from within the body (i.e., interoception). The theory of embodied predictive coding suggests that feelings indicate a process of continually updating self-generated predictions about the probable causes of sensory input^4–6^. This suggests new opportunities for intervening on human affect through the controlled stimulation of interoceptive signals and somatosensation. The actuation of human emotion via bodily stimulation has been suggested as an ideal case for investigating interoception and designing clinical interventions^7,8^. Here, we investigated embodied predictive coding by modulating bodily sensations underlying aesthetic chills (i.e., goosebumps, psychogenic shivers), using a device modulating cold and shiver sensations down the spine characteristic of this peak emotion. Our study is based on the paradigm of interoceptive illusions or misattribution of arousal^9^, wherein external stimulation, such as temperature change or increased heart rate, modulate the interoceptive inferences underlying decision-making to trigger predictable behavioral change^10,11^.

Interoceptive illusions have attracted some attention in recent years as experimental tools for interoceptive neuroscience^11^. In a seminal study, Valins et al. demonstrated that increasing heart rate via mere physical exercise facilitates romantic attraction to confederates^9^. This is attributed to the misinterpretation of increased heart rate as due to attraction, as opposed to exercise^12^. Other experiments have manipulated various forms of physiological emotional feedback, including facial muscles^13–15^. The modulation of interoceptive inferences through the controlled simulation of emotional somatic markers has shown promising results in reducing stress^16^ and fear^17^. Recent evidence in favor of the model of embodied predictive coding suggests a causal relation between altering interoception and corresponding changes in emotion^10,11^. Modulating somatic markers and altering interoceptive inference during the phase of emotion experience may allow us to disentangle historical key questions in the field of affective neuroscience, namely the problem of causality and dynamics in emotional networks, and in turn suggest new interventions for somatic disorders^18^. However, interoceptive illusion studies are generally based on physiological markers lacking in universality. For example, a smile conveys various meanings across cultures and communities^19^. In such cases most studies failed to replicate and thereby scientific paradigms for bottom-up influences of the body on higher cognitive processes remain opaque^20^.

The aim of this study is to test the possibility of modulating cognition from the bottom-up (from body to mind), through actuation of a device designed to simulate and stimulate aesthetic chills. Aesthetic chills are a somatic marker for individual emotional peaks^21^ described as a highly pleasurable tingling sensation down the spine^22^. These are most often elicited by music^23,24^, but also poetry^25^, scientific insights^26^ or social rituals^26^. These events have deep significance to individuals, often related to life changing experience such as the overview effect^27^ and transformative experiences^28,29^, and are sometimes accompanied by tears^29^. In terms of physiology, chills have a specific cardiac signature^30^ and are primarily accompanied by an increase of phasic electrodermal activity and respiration depth^31^. Chills have been described as a physiological marker of salience^26^, a self signalling mechanism allowing the cognitive system to orient its attentional resources toward evolutionary relevant stimuli^26,32^. Furthermore, negatively valenced aversive chills have also been observed in relation to aversive auditory stimulation or traumatic stimuli^33,34^. Both positive and negative chills engage neural populations coding for salience such as amygdaloid complex, ventromedial prefrontal cortex and the nucleus accumbens^22,34^. Chills have been related to various complex emotional states such as awe^35,36^, insight^37^, prosocial emotions^38^, and being moved^25^. Their downstream effects include positive effects on social cognition^37,39^ and promotion of altruistic behavior^38,40^. Schoeller and Perlovsky have put forward a theory of chills as a satiation of an internal drive for knowledge^26,41^. Chills would thus correspond to a sudden acceleration of learning^42–45^ described formally in terms of an event when the rate of change of a learning function tends towards zero^26,37,39,41^. This account is coherent with current accounts of emotional valence in terms of error dynamics^46,47^. Crucially, and even though their prevalence across human populations is still an open question, psychogenic shivers seem to present a high degree of universality, making them a useful somatic marker for affective neuroscience in light of their myriad emotional links^38,43,44,48,49^.

As a salient interoceptive inference and somatic marker of a peak emotion, chills are an ideal case for disentangling the role of conscious and unconscious evaluations of bodily signals during emotional processes. Here, we tested whether we could artificially induce peak emotion by stimulating chills with spatial and temporal precision using a wearable prosthesis designed for this purpose. The prosthesis delivers cold and vibration stimulus at critical reference points of an audiovisual stimulus of awe-provoking images of the planet Earth from above accompanied with a chills-eliciting speech and music (see “Methods” section and “Supplementary Information”). Through this controlled stimulation, we aimed to amplify the sensory signals serving as a basis of the feeling of chills, and replicate some of the downstream effects. We therefore predicted that the device would increase the frequency and intensity of chills, and trigger changes in terms of pleasure and social cognition, which are known downstream effects for aesthetic chills^40,41^. To get at the underlying psychology driving the positive relationship between aesthetic chills and altruism, we chose to distinguish between the two broad categories of emotional and cognitive social processes which are likely factors: emotional contagion (i.e., the degree to which one party shares the feelings of another, separate from the ability to accurately intuit what another person is feeling) and cognitive empathy (i.e., the degree to which one share’s somebody’s viewpoint)^50–52^.

## Results

**Figure 1:**
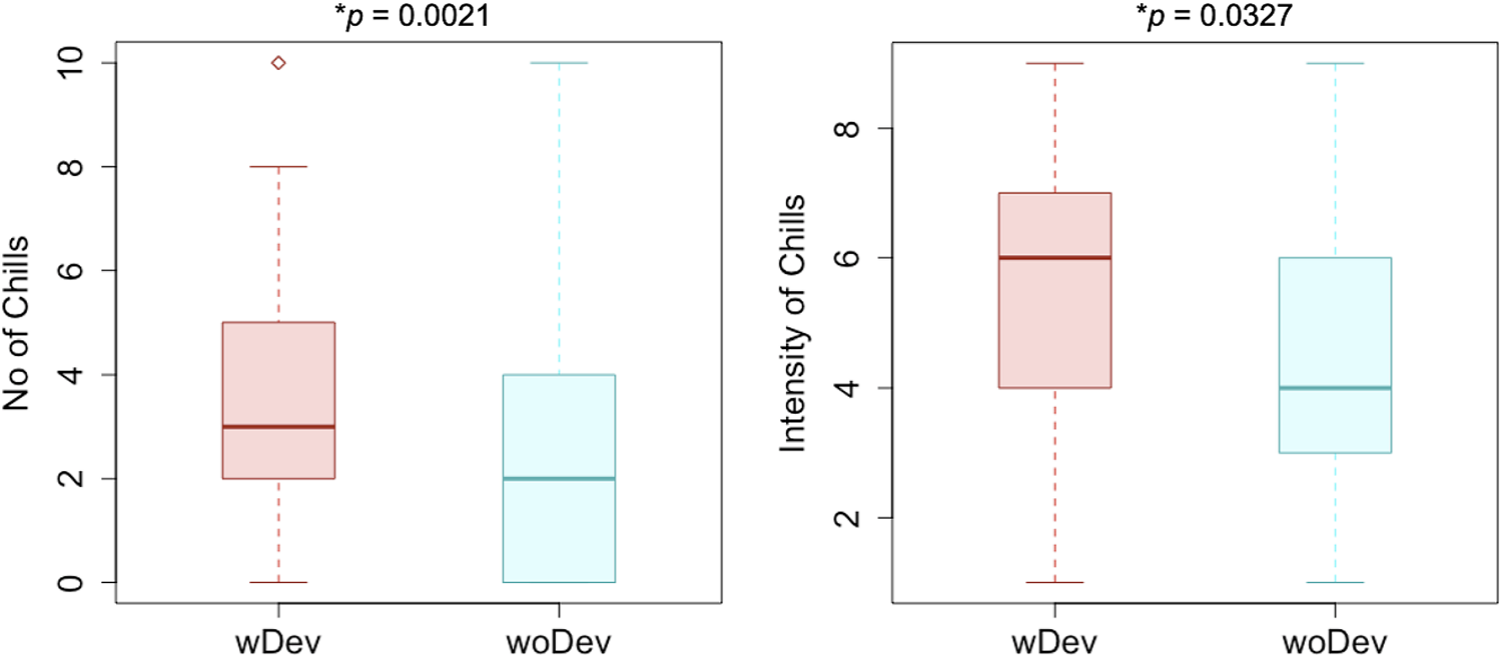
Number and Intensity of chills per conditions: participants wearing the device reported experiencing significantly greater number and intensity of chills during the experience with the device than during the experience without the device.

Subjects (n = 21) reported number of chills on a range from 0–10. Participants experienced a higher number of chills while wearing the device (M = 4.00; STD = 2.90) than without the device (M = 2.76; SD = 2.86) (Fig. 1). A pairwise one-tailed t-test revealed that the device significantly increases the frequency of chills (t = 3.2274, df = 20, p = 0.0021) and Shapiro–Wilk normality test (W = 0.96964, p = 0.7251) does not reject the null hypothesis of data normality. Participants also reported a higher intensity of chills while wearing the device (M = 5.62; SD = 2.44) than without the device (M = 4.33, SD = 2.57) (Fig. 1). Since reported intensity is a likert scale non parametric data, we used a pairwise one-tailed Wilcoxon test to assess the difference and found a significant difference in chills intensity across groups (V = 104, p = 0.0327) (Table 1). Hence, at any significance level, this test rejects the null hypothesis of independence between conditions and chills frequency and intensity.

**Table 1:** Comparison of the intensity of chills, sharing of the speaker’s feelings, sharing of the speaker’s viewpoint, and perceived pleasure with and without the device.

Measure	With device	Without device	Pairwise one-sided Wilcoxon
Intensity of chills	5.62 ± 2.44	4.33 ± 2.57	V = 104, p* = 0.0327
Pleasure	7.66 ± 1.28	6.95 ± 1.66	V = 38.5, p* = 0.0321
Cognitive empathy	8.33 ± 1.15	8.09 ± 1.79	V = 18.5, p = 0.5
Emotional contagion	8.00 ± 1.73	7.19 ± 2.40	V = 76, p* = 0.0147

We then considered the physiological data from the camera and cardiac sensors. Some of the physiological data was lost after collection and the remaining data did not reveal any significant difference across groups. We first examined the camera data for the remaining sample (N = 11), and found a higher count of smiles in the recordings for the participants wearing the device (M = 1.18; STD = 1.78) than participants without (M = 0.27; STD = 0.65). A pairwise two-sided Wilcoxon test revealed that this difference is not significant (V = 51, p = 0.3804). We then analysed the heart rate data for the remaining sample (N = 12), and found an increase in the LF/HF ratio for participants with the device (M = 2.23; STD = 4.18) than without (M = 1.75; STD = 2.98). A pairwise two-sided Wilcoxon test revealed this difference is not significant (V = 12.5, p = 0.2228). Hence, these marginal differences do not allow us to reject the null hypothesis for physiological differences in facial expression or heart rate frequency data across groups.

**Figure 2:**
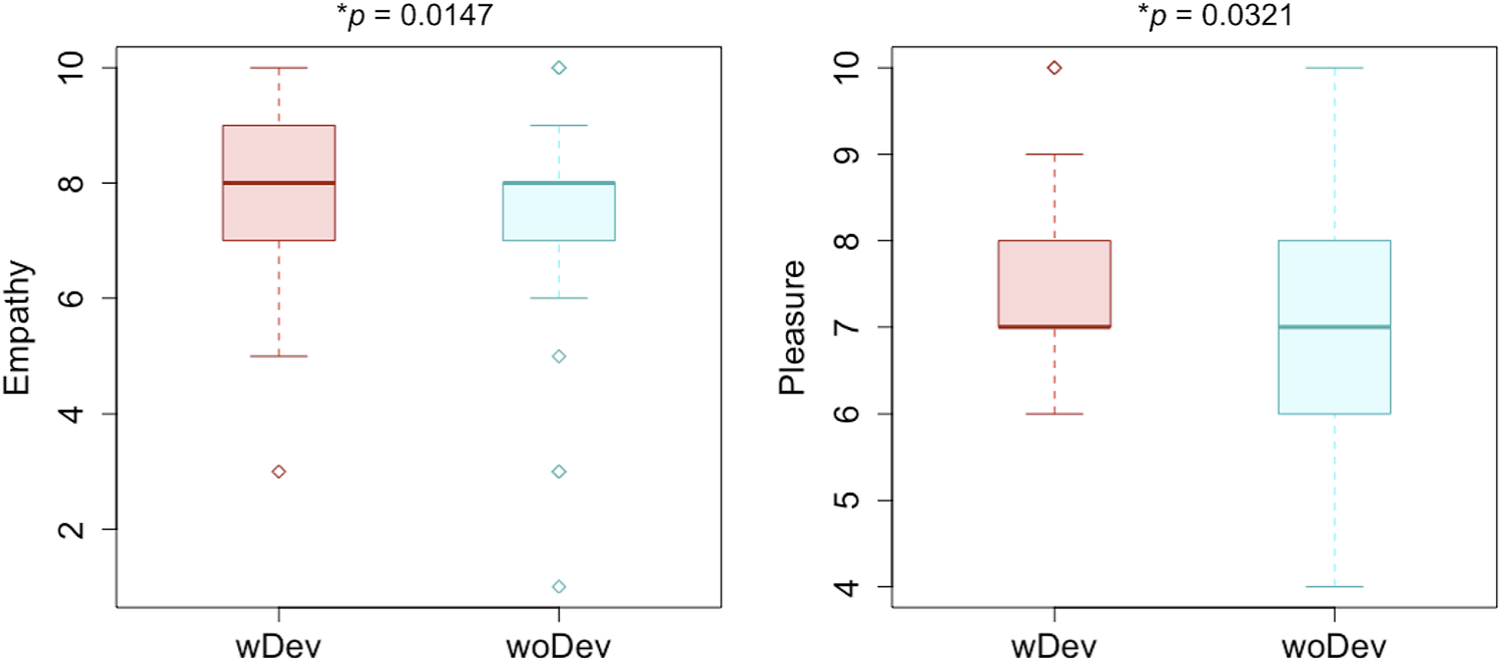
Empathy and pleasure of chills per conditions: participants wearing the device reported experiencing significantly greater emotional contagion and more pleasure during the experience with the device than during the experience without the device.

We then analyzed the downstream effects of the device on pleasure and empathy as reported by participants (see Table 1 and Fig. 2). Participants reported greater emotional contagion while wearing the device (M = 8.00 SD = 1.73) than without the device (M = 7.19; SD = 2.40). A Shapiro–Wilk normality test rejected the null hypothesis of of data normality (W = 0.79563, p = 0.0006), therefore we used a non-parametric pairwise one-sided Wilcoxon to test the difference across group, which confirmed a significant difference in empathy across groups (V = 76, p = 0.0147). We also analyzed reports of cognitive empathy (i.e., shared viewpoints). Participants wearing the device reported sharing the speaker’s viewpoint (M = 8.33; STD = 1.15) more than when viewing the stimulus without the device (M = 8.09; STD = 1.79). However, a pairwise one-sided Wilcoxon test revealed the difference to be not significant (V = 18.5, p = 0.5). Participants with the device also reported a greater amount of pleasure in the viewing experience (M = 7.66; SD = 1.28) than those without the device (M = 6.95; SD = 1.66). As this data does not seem to follow a normal distribution (W = 0.81652, p = 0.0012), we used a pairwise one-sided Wilcoxon test to test for differences across groups, which revealed a significant difference (V = 38.5, p = 0.032). Hence, we can reject the null hypothesis of independence between conditions and stimulus-elicited pleasure and emotional contagion, but not for cognitive empathy across groups.

**Figure 3:**
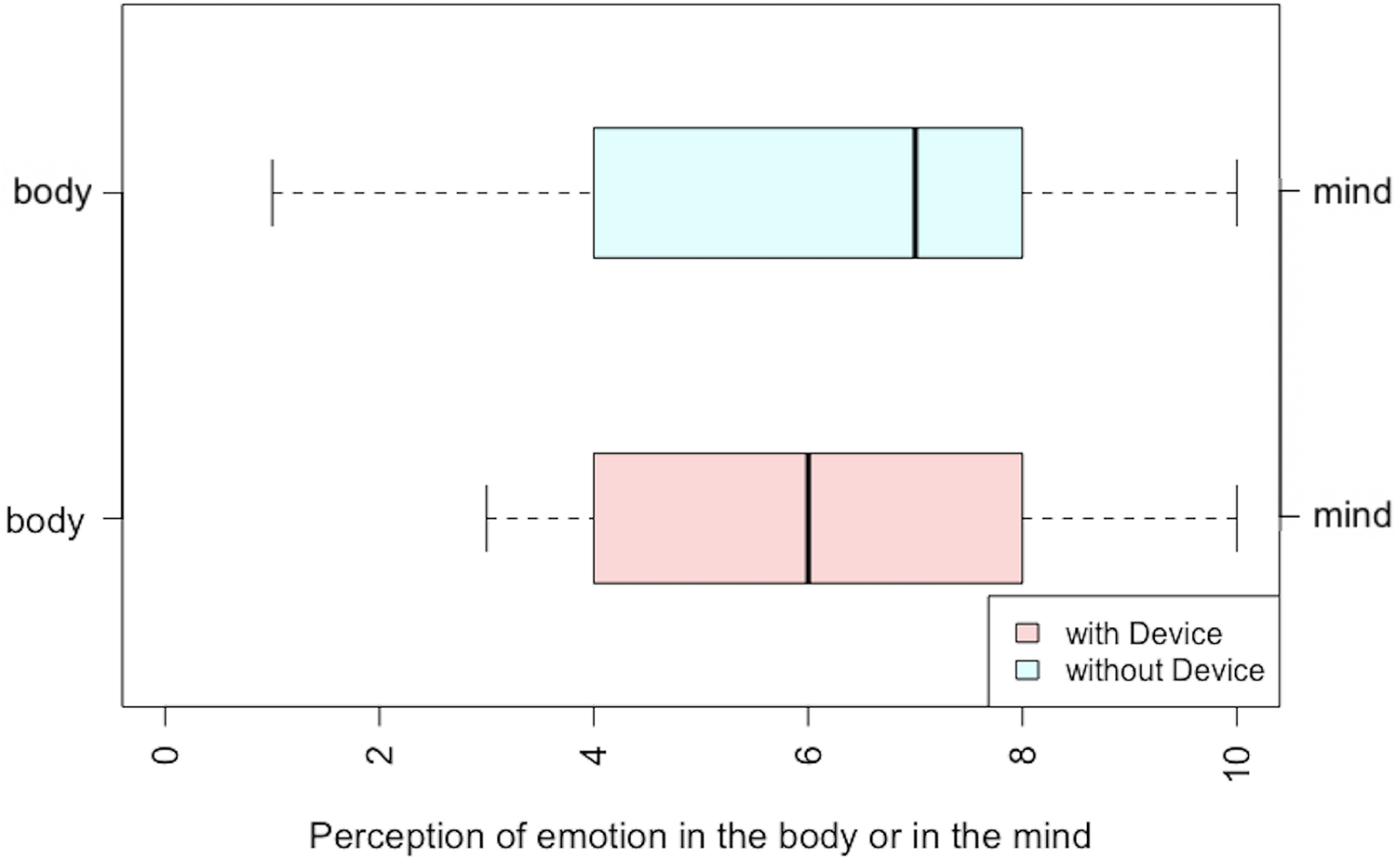
Perception of the felt emotion (in the body or in the mind) reported by the participants.

**Figure 4:**
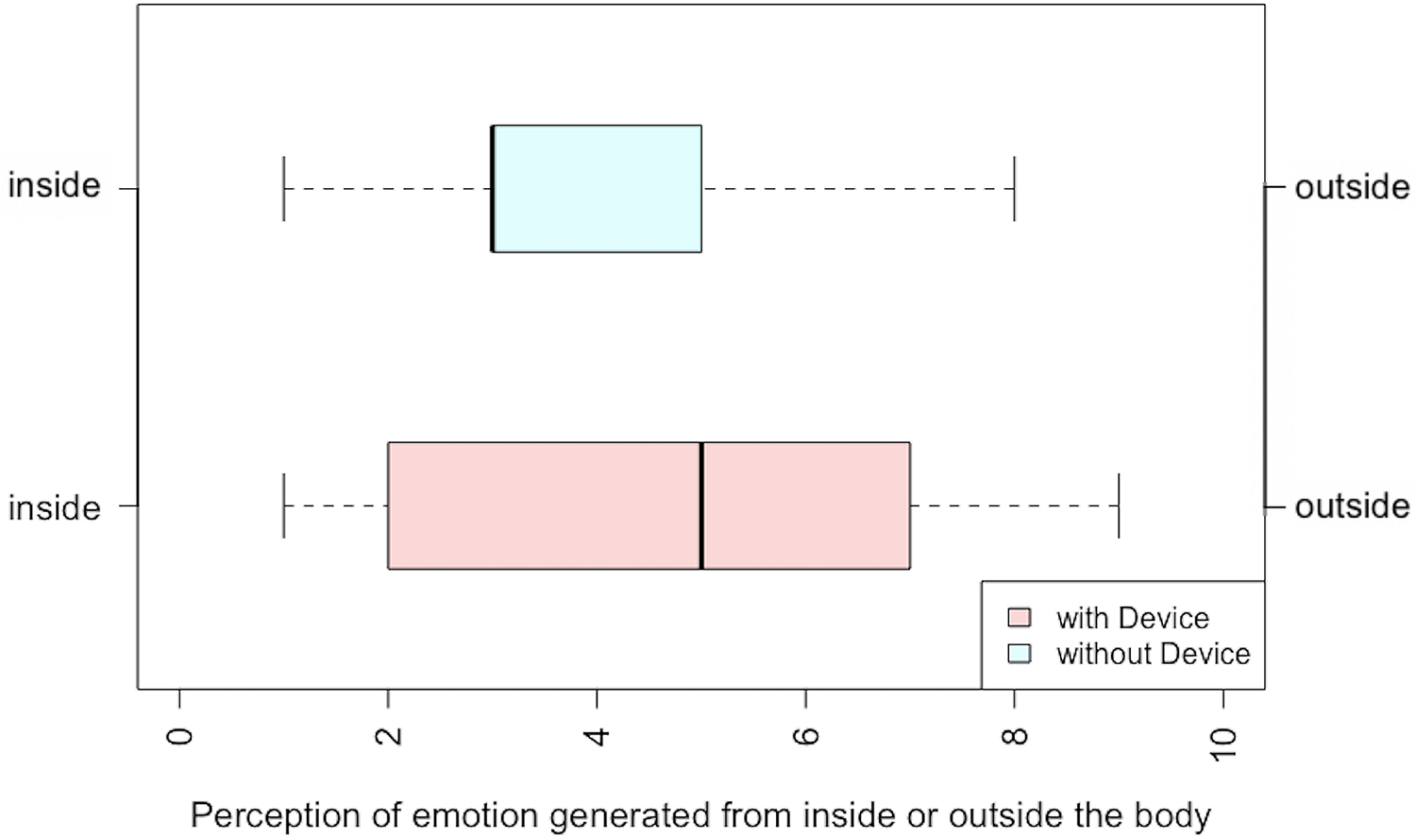
Perception of emotion generation (inside the body or outside the body) reported by the participants.

To understand the relationship of the body-worn device to reported emotions, we further investigated participant’s subjective bodily localization of emotion and perceived point of generation of the felt emotion (see Table 2). Participants reported whether their emotions were felt in the body or mind (on a 0–10 likert scale where 0 corresponded to feeling in the body and 10 to in the mind, Fig. 3) and also whether the emotion was generated internally or externally (where 0 corresponded to reports of emotion generated inside the body and 10 to generated outside the body, Fig. 4). We found no significant differences across conditions. Lastly, we asked participants to rate their confidence regarding their understanding of the speech (on a likert scale from 0 to 10 where 1 corresponded to not confident at all and 10 corresponded to extremely confident). We found no significant difference across conditions in reported confidence.

**Table 2:** Comparison of the viewer’s confidence regarding understanding of the stimulus, perception of emotion generation inside or outside the body, and perception of emotion felt in the body or the mind across device conditions.

Measure	With device	Without device	Pairwise two-sided Wilcoxon
Confidence	8.00 ± 1.73	8.33 ± 1.62	V = 14, p = 0.1738
Emotion generated in the body or the mind	5.95 ± 2.27	6.14 ± 2.41	V = 48, p = 0.5059
Emotion generated inside or outside the body	4.57 ± 2.80	4.05 ± 2.03	V = 64.5, p = 0.4682

## Discussion

This study investigated the artificial induction of aesthetic chills using a wearable prosthesis. We found that stimulation from the Frisson device significantly increased the frequency of chills experienced by the participants, as well as ratings of chill intensity. When examining the downstream effects of these augmented chills, we found that participants also reported significantly more empathy towards the speaker presented in the audiovisual stimulus (in terms of emotional contagion i.e. sharing the speaker’s feelings to a greater degree), and a significant increase in ratings of how pleasurable the video-watching experience was. We found that across conditions participants did not report significantly different confidence regarding understanding the stimulus, suggesting the downstream effects mentioned above are not tied to the percept of better understanding of the stimulus speech or the pleasure of understanding. Results suggest that artificially inducing chills may facilitate their downstream effects on social cognition^40^. Results further lend some credence to the predictive processing model of emotion as predicted interoceptive input^4–6^, as subjects with interoception of chills (synthetic or organic) were more likely to report the emotions associated with organic chills. Interestingly, subjects wearing the Frisson device did not report a significant difference in embodied localization or point of generation of emotions, suggesting the device itself did not play a large role in the reflection on felt emotion, though the chill it created indeed did. This in turn raises opportunities for future research into affective neuroscience and interoceptive inferences associated with somatosensory stimulus.

The neuroscience of emotion has identified close correlational links between states of physiological and emotional arousal. These include relationships between pupil size, galvanic skin response, or heart rate with affective arousal^53^. Yet historically, multiple theories have posited an active, causal as opposed to correlational role for bodily states in the creation of emotion. William James famously hypothesized that felt emotion is “not a primary feeling, directly aroused by the exciting object or thought, but a secondary feeling indirectly aroused; the primary effect being the organic changes in question, which are immediate reflexes following upon the presence of the object”^54^. The present results motivate further testing for causal rather than correlational links between changes in bodily state and changes in affective state^55^. The complex interplay of cognitive and physiological factors involved in emotional processes makes order and causality difficult to disentangle in affective neuroscience^56^. Experiments in misattribution of arousal, wherein experimenters modulate a subject’s physiology in order to alter cognition, offer an initial probe^9^. Emotion prosthesis and somatosensory interfaces such as our Frisson device open new pathways for affective neuroscience, by allowing the augmentation or modulation of interoceptive inferences. New research tools allowing researchers to actuate embodied emotion as causal probes beyond the study of correlations are necessary for an effective and applied affective neuroscience. This exploratory study raises a number of significant questions for further research, including what neural mechanisms underlie the integration of artificial sensory information and how organic endogenous sensations are processed differently from exogenous synthetic stimulations.

However, this study must also be understood in the light of its limitations. First, the sample sizes in this exploratory study are limited in scope and diversity. With a subject n = 21, the current study was likely powered to detect only very large effects and any effect sizes reported may be overestimated. This exploratory study should be replicated on a larger sample in a wide range of ecological settings including culturally relevant stimuli (which may vary cross-culturally) and across heterogenous human populations. Second, a next iteration of the device should allow for a sham back-worn device to limit expectation effects in the control condition to a strict minimum. Third, emotional contagion is only one element in the complex construct of empathy, and one could ‘catch’ feelings from a stimulus even while misinterpreting the emotion displayed therein. Finally, as chills appear to be independent of valence, further study should examine the potential of the device on modulating the downstream effects of negative chills as well.

Emotion prosthetics and somatosensory interfaces offer new possibilities for affective neuroscience inducing human emotions from the bottom-up, modulating their associated metacognitive judgments (i.e., feelings) and downstream effects (i.e., action tendency) through interoceptive technology. The constant feedback loop between actual and expected sensations during interoceptive processing allows for intervention on higher cognitive functioning through controlled stimulation of body signals. Surprisingly little research has been devoted to the topic of how technology may be used to enhance or generate emotion from modulating interoceptive inferences and somatic markers (i.e., somatosensory interfaces). If confirmed in further studies, these results suggest that in the long term, emotion prosthetics and somatosensory interfaces may provide novel non-invasive tools for understanding interoception-related disorders as well^18^.

## Methods

We used a within-subject design to investigate whether one can artificially modulate interoceptive inferences underlying aesthetic chills, their felt frequency and intensity. The hypothesis was that both psychological and physiological responses would be different between with and without the Frisson stimulation device. Experiments were conducted using self-report, a muscle-bend sensor allowing for silent report of chills experience, image capture for facial expression analysis, physiological sensors (heart rate and skin conductance) for measuring physiological changes concurrent with frisson, and both quantitative and qualitative surveys.

### Protocol

The participant entered the laboratory between 12:00 and 4:00 p.m., sat in front of a computer monitor, and was provided with a consent form. The study and consent form provided were approved by the Institutional Review Board, the Committee on the Use of Humans as Experimental Subjects at MIT. Participants were told that the study examined the relationship between temperature and attention, and were specified the definition of aesthetic chills as psychogenic waves of cold as opposed to external cold stimulations from device, and asked to report only the former. The stimulation device was placed on their back and sensors were wrapped around the middle phalanges of their right hand’s index and middle finger. Participants were told to clench their hand if they experienced aesthetic chills at any time during the film, such that the handworn sensor could collect a count of chills. Next, each participant was exposed to a calming film of a cold landscape (ice mountains and cascade) for 90 s to control for stress baseline and prime the subject with the concept of cold. Next, the chill-eliciting video stimulus began, entailing a 213 s long speech and introductory message about the film content. Artificial chills were delivered at timecodes 2:43, 3:52 and 4:03. The same video viewing procedure was done with or without the Frisson back-worn stimulation device, depending on condition, but participants wore hand-worn physiological sensors regardless of condition. We chose to remove the back-worn device in the control condition in order to avoid any transient cold temperature from the device. To mitigate expectation effects differing across conditions, we presented the chills device as both the sensor on the back and actuator on the wrist, allowing us to remove the back-worn part of the device in the control condition but maintain that ‘the device’ remained on their body. We collected subjective data in the form of surveys after each condition which asked questions such as the frequency of chills experienced by the participant, the intensity of the chills experienced, degree to which subjects shared the speaker's viewpoint, and the degree to which subjects shared the speaker's feelings. The subjective questions were asked on a likert scale of 0–10 (see “Supplementary Information” for further details about the procedure, stimulus and questionnaire). Once the experiment was finished, the experimenter disconnected the sensors and provided the subject with a questionnaire. Finally, the subjects were thanked for their participation and fully debriefed. Each session lasted about 20 min.

### Participants

A total of 21 students participated in the experiment (N females = 7, N males = 14, M age = 27, STD = 6.5). Sample size was determined based on a compiled exhaustive database of aesthetic chills research (see “Supplementary Information”). We determined that an acceptable sample size for an exploratory study of this kind ranged from 20 to 50. On their arrival at the laboratory waiting room, they were randomly assigned to one of the experimental conditions. Following a within subject design, each participant took part in both experimental conditions: once with and once without the device, each time exposed to the same audiovisual stimulus. The order was counterbalanced to account for learning effects.

## Materials

The materials used in the experiment are in the following section.

### Stimulus

The audiovisual stimulus was presented using a standard computer screen and headphones with a fixed volume. Following on a preliminary study using responses to a survey inquiring into the properties of chill-eliciting situations (see^41^) and a software for searching YouTube videos in terms of their density of chills-related comments, we designed the stimulus combining two modalities (audio and visual) likely to trigger chills in the studied population. The content for each modality was identified using a tool specifically developed for this purpose^42^, a software to automatically retrieve online content triggering chills on YouTube (as measured by the prevalence of chills related comments by users in the platform). Our search yielded a number of videos and we combined audio (speech and music) and visual components (images of earth) from the content most likely to trigger psychogenic shivers in the target population (the software sorts through networks of videos reflecting the human populations uploading them). Film audio tracks are more powerful than music in eliciting piloerection, a common marker of aesthetic chills^31^. The visual stimulus was a 4 K satellite view of planet Earth, a stimulus known to trigger awe and the overview effects likely to trigger chills^27,35,36^. As a voice over, we used a speech excerpt of Charlie Chaplin from the movie ‘The Great Dictator’ accompanied by a Hans Zimmer musical soundtrack. The film was subtitled in English. The introductory message that preceded the film was a block of text presenting the following: “What you are about to hear is the voice of Charlie Chaplin from the middle of World War II. For his entire career, he had been a silent actor. But in 1940, he decided to speak out. This is his message of hope”. For further discussion on contents likely to elicit chills, refer to^8,26,37,39,41–45^.

### Actuator

**Figure 5:**
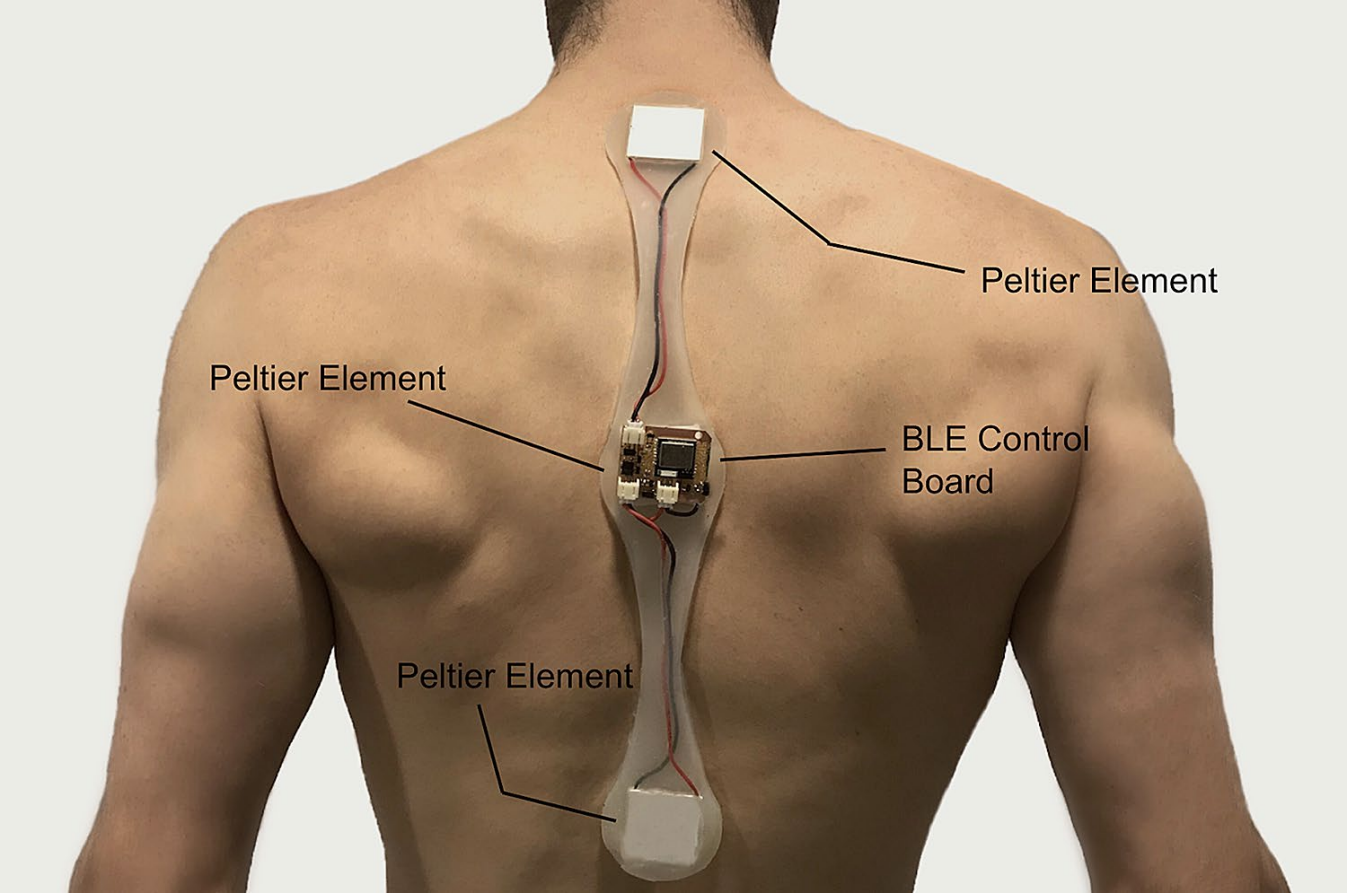
The Frisson prosthesis: a device delivering thermal feedback in a manner closely resembling to the internal chill, a traversing cold temperature from top to bottom.

A prototype was developed to deliver a thermal and vibrotactile feedback down the spine and imitate the sensation of chills. The device consisted of three peltier elements at different spatial locations: top of the back, one on middle and one on the lower back, and a BLE enabled control circuit board. We tested the device in a series of preliminary experiments, and improved the design based on participant feedback to reproduce the sensation of chills (see “Supplementary Information”). The final device delivered thermal feedback in a manner closely resembling the internal chill, a traversing cold temperature from top to bottom for a period of 3 s and a short burst of tingling vibration at the top of the back for 1 s (Fig. 5). The device was powered by a 2S 7.4 V 500 mAH Lipo battery, and was cast in silicone for easy attachment to the back of the participants. An Android mobile application was developed to activate the device with specific timing, delivering the chills in synchrony with the video stimulus.

### Sensors and software

Physiological data was collected using hand worn sensors from the Dormio device^57–59^. We collected heart rate using a Sparkfun Pulse Sensor Amped PPG sensor from the middle finger and electrodermal activity using dry electrodes from the wrist. We also attached a flexion sensor on the index finger and asked participants to clench their index finger when experiencing a chill sensation. The data was sampled at 100 Hz. All the data was collected using Bluetooth to a PC. Facial expressions data was recorded using a camera mounted over the screen and stored in a SD card.We used Affdex SDK for detecting facial expressional from recorded video at 30fps. Affex SDK detected 12 relevant metrics from the video. The metrics were ‘smile’, ‘anger’, ‘valence’, ‘browFurrow’, ‘noseWrinkle’, ‘joy’, ‘surprise’, ‘browRaise’, ‘upperLipRaise’, ‘mouthOpen’, ‘eyeClosure’, ‘cheekRaise’.

### Ethics

The experiment is in compliance with the Helsinki Declaration. The study was approved by the Committee on the Use of Humans as Experimental Subjects at MIT. All participants gave their voluntary informed consent and we followed the Ethics Code of the American Psychological Association. All participants were informed about the purpose of the research, about their right to decline to participate and to withdraw from the experiment and about the limits of confidentiality. We also provided them with a contact for any questions concerning the research and with the opportunity to ask any questions regarding the phenomenon under study (aesthetic chills) and receive appropriate answers. All participants reacted positively to the experiment and were thankful for the opportunity to learn about the phenomenon. Following the conclusion of the experiment, we engaged subjects in many discussions on the potential abuses inherent in technologies which are built to alter human emotion. As these devices become more subtle and more advanced, these issues will come clearly to the fore. We build our devices for impossibility of surreptitious use (i.e. subjects could not fail to notice their application) but are aware that continued conversation around ethical use of these devices and public education around potential for influence are necessary and fruitful.

### Reviewer disclosure

Following the standard reviewer disclosure request endorsed by the Center for Open Science^60^, We confirm to have reported all measures, conditions, data exclusions and how we determined our sample sizes.

## Supplementary information

Supplementary Information.

## Data Availability

Supporting data is available at public repository: https://doi.org/10.7910/DVN/E4ZYOT.
