# Supplementary material for: Augmenting aesthetic chills using a wearable prosthesis improves their downstream effects on reward and social cognition
Source: Sci Rep. 2020 Dec 10;10:21603. doi: 10.1038/s41598-020-77951-w (PMC7728802; doi:10.1038/s41598-020-77951-w)
Supplement: Supplementary file 1 — Supplementary Information. [file 41598_2020_77951_MOESM1_ESM.pdf]

Supplementary information: Augmenting aesthetic chills using a wearable prosthesis improves their downstream effects on reward and social cognition

Haar, A.J.H. [1]*, Jain, A.[1], Schoeller F. [1,2], Maes, P.[1]

[1] Fluid Interfaces Group, Media Lab, Massachusetts Institute of Technology, Cambridge, USA.

[2] Institut de Psychologie, Université Paris-Descartes, France

*correspondence: adamjhh@mit.edu

1. Preliminary studies

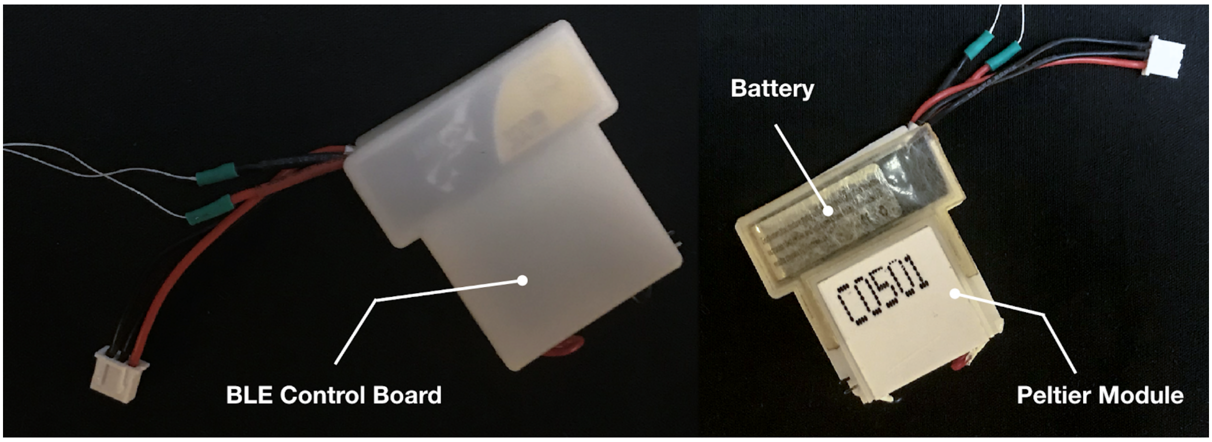

Figure 1. Preliminary frisson device using a single peltier module.

1.1 Methods

To test and improve the effects of the preliminary device (Figure 1), we conducted a preliminary study, whereby 52 subjects (International interdisciplinary students (American, European and Asian) from the Harvard Summer School at the Centre for Research and Interdisciplinarity) were distributed into three conditions. 23 subjects were exposed to treatment (chills stimulus alone), 19 to control (film about everyday life of student), and 10 subjects (3 females, M age=40, STD=8) were exposed to chill eliciting stimulus and received an additional chills stimulus from the first version of the actuator placed on their right arm (actuator).

1.2 Results

We build a comparison table (Table 1). In terms of pleasure, there is an extremely significant difference for treatment and actuator at $p << .01$, in comparison to control, the result is

significant at $p < .05$. In terms of pleasure, highly significant difference between treatment and actuator ($p << .01$). We test for the difference between treatment and actuator in terms of chills. The Fisher exact test statistic value is 0.3698. The result is not significant at $p < .05$.

Table 1. Subjective pleasure and relaxation across groups.

Group	N	Chills	% chills	Pleasure	Relax
Treatment	23	8	34,78	7,52	7,61
Control	19	0	0,00	6,11	6,16
Actuator	10	6	60,00	4,89	5,50

1.3 Discussion

Actuator seems reliable in eliciting chills. However, arms may not be the optimal location. No significant difference in terms of chills (which we attribute to small sample size). However, these chills are less pleasurable and relaxing than all conditions we tested. We attribute low pleasure to user experience and low relaxation to white coat effect and the preliminary nature of the protocol. All in all these results are encouraging but demand the user experience to be improved. New locations should be tested to build a gradient.

2. Methods

We used a within-subject design to investigate whether one can artificially modulate interoceptive inferences underlying aesthetic chills, their felt frequency and intensity. The hypothesis was that both psychological and physiological responses would be different between with and without the Frisson stimulation device. Experiments were conducted using self-report, a muscle-bend sensor allowing for silent report of chills experience, image capture for facial expression analysis, physiological sensors (heart rate and skin conductance) for measuring physiological changes concurrent with frisson, and both quantitative and qualitative surveys.

2.1. Protocol

The participant entered the laboratory between 12:00-4:00 pm, sat in front of a computer monitor, and was provided with a consent form. Participants were told that the study

examined the relationship between temperature and attention, and were specified the definition of aesthetic chills as psychogenic waves of cold as opposed to external cold stimulations from the device, and asked to report only the former. The stimulation device was placed on their back and sensors were wrapped around the middle phalanges of their right hand's index and middle finger. Participants were told to clench their hand if they experienced aesthetic chills at any time during the film, such that the handworn sensor could collect a count of chills. Next, each participant was exposed to a calming film of a cold landscape (ice mountains and cascade) for 90s to control for stress baseline and prime the subject with the concept of cold. Next, the chill-eliciting video stimulus began, entailing a 213s long speech and introductory message about the film content. Artificial chills were delivered at timecodes 2:43, 3:52 and 4:03. The same video viewing procedure was done with or without the Frisson stimulation device, depending on condition, but participants wore hand-worn physiological sensors regardless of condition. We collected subjective data in the form of surveys after each condition which asked questions such as the frequency of chills experienced by the participant, the intensity of the chills experienced, degree to which subjects shared the speaker's viewpoint, the degree to which subjects shared the speaker's feelings, confidence of understanding the video and location of perception of emotion. The subjective questions were asked on a likert scale of 0-10 (see supplementary materials for further details about the procedure, stimulus and questionnaire). Once the experiment was finished, the experimenter disconnected the sensors and provided the subject with a questionnaire. Finally, the subjects were thanked for their participation and fully debriefed. Each session lasted about 20 min.

2.2. Participants

A total of 21 students participated in the experiment (N females= 7, N males =14, M age=27, STD=6.5). Sample size acceptable range was determined based on a compiled exhaustive database of aesthetic chills research (see below). On their arrival at the laboratory waiting room, they were randomly assigned to one of the experimental conditions. Following a within subject design, each participant took part in both experimental conditions: once with and once without the device, each time exposed to the same audiovisual stimulus. The order was counterbalanced to account for learning effects.

Database of chills research

We constructed an exhaustive database of existing chills research. The RDF semantic web structured intelligent DB contains all existing academic literature on chills (N~40). All papers are listed in the Index tab. A user interface can be accessed at http://51.68.79.244:4001. There one can select various properties and construct queries for the DB (e.g., listing all studies "on musc" conducted with "ECG"). If needed login/password: "guest". The compiled list of all aesthetic chills experiments are also available at the following url: **https://bit.ly/2I8oFlV**

Table 2: Articles selected from chills database to determine the sample size for this study.

DOI	Sample size
https://pubmed.ncbi.nlm.nih.gov/27540366/	30
https://pubmed.ncbi.nlm.nih.gov/26966157/	20
https://www.pnas.org/content/98/20/11818	10
https://journals.plos.org/plosone/article?id=10.1371/journal.pone.0130117	58
https://www.ncbi.nlm.nih.gov/pmc/articles/PMC5384201/	32
https://www.frontiersin.org/articles/10.3389/fpsyg.2014.01215/full	22
https://www.sciencedirect.com/science/article/pii/S1389041717300232	30
https://www.sciencedirect.com/science/article/pii/S0033298417300791?via%3Dihub	30

2.3. Materials

The materials used in the experiment are in the following section.

2.3.1. Stimulus

The audiovisual stimulus was presented using a standard computer screen and headphones with a fixed volume. Following on a preliminary study using responses to a survey inquiring into the properties of chill-eliciting situations [32] and a software for searching YouTube videos in terms of their density of chills-related comments, we designed the stimulus combining two modalities (audio and visual) likely to trigger chills in the studied population. Film audio tracks are more powerful than music in eliciting piloerection, a common marker

of aesthetic chills [4]. The visual stimulus was a 4K satellite view of planet Earth, a stimulus known to trigger the overview effects likely to trigger chills [37]. As a voice over, we used a speech excerpt of Charlie Chaplin from the movie 'The Great Dictator' accompanied by a Hans Zimmer musical soundtrack. The film was subtitled in English. The introductory message that preceded the film was a block of text presenting the following: "What you are about to hear is the voice of Charlie Chaplin from the middle of World War II. For his entire career, he had been a silent actor. But in 1940, he decided to speak out. This is his message of hope". For further discussion on contents likely to elicit chills, refer to [30].

The stimulus is available at the following URL: **https://bit.ly/2VwBFnN**

2.3.2. Actuator

A prototype was developed to deliver a thermal and vibrotactile feedback down the spine and imitate the sensation of chills. The device consisted of three peltier elements at different spatial locations: top of the back, one on middle and one on the lower back, and a BLE enabled control circuit board. We tested the device in a series of preliminary experiments, and improved the design based on participant feedback to reproduce the sensation of chills. The final device delivered thermal feedback in a manner closely resembling the internal chill, a traversing cold temperature from top to bottom for a period of 3s and a short burst of tingling vibration at the top of the back for 1s (Figure 2). The device was powered by a 2S 7.4V 500mAH Lipo battery, and was cast in silicone for easy attachment to the back of the participants. An Android mobile application was developed to activate the device with specific timing, delivering the chills in synchrony with the video stimulus.

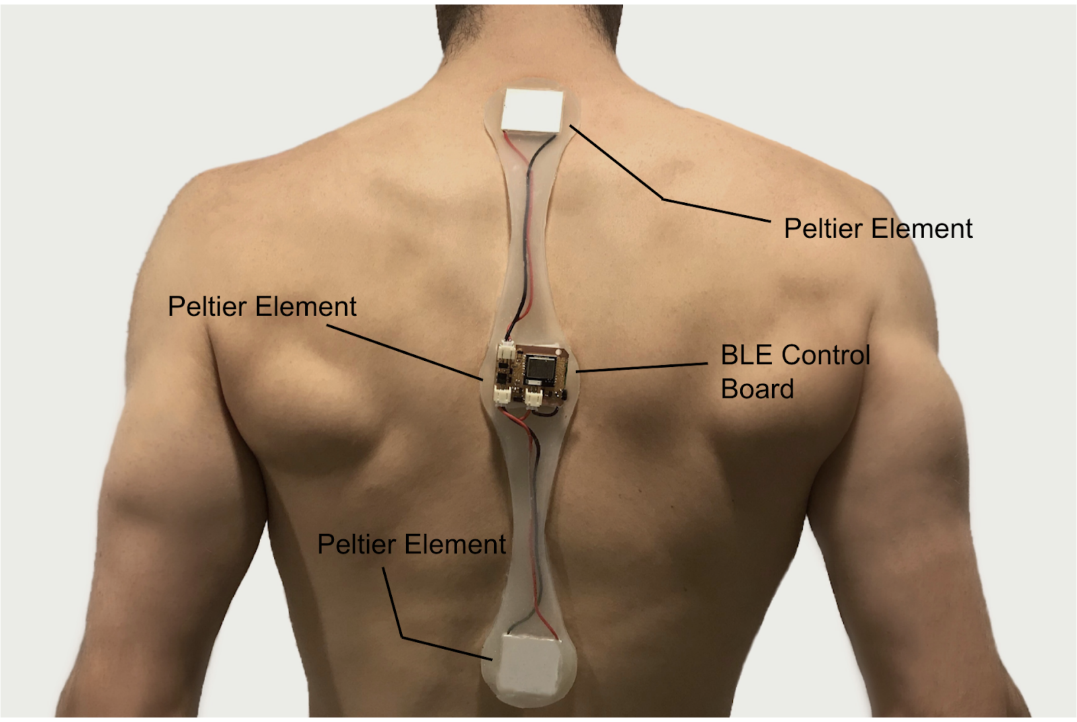

Figure 2. The Frisson prosthesis: a device delivering thermal feedback in a manner closely resembling the internal chill, a traversing cold temperature from top to bottom.

2.3.3. Sensors and software

Physiological data was collected using hand worn sensors from the Dormio device [14]. We collected heart rate using a Sparkfun Pulse Sensor Amped PPG sensor from the middle finger and electrodermal activity using dry electrodes from the wrist. We also attached a flexion sensor on the index finger and asked participants to clench their index finger when experiencing a chill sensation. The data was sampled at 100Hz. All the data was collected using Bluetooth to a PC. Facial expressions data was recorded using a camera mounted over the screen and stored in a SD card.We used Affdex SDK for detecting facial expressional from recorded video at 30fps. Affex SDK detected 12 relevant metrics from the video. The metrics were **'smile', 'anger', 'valence', 'browFurrow', 'noseWrinkle', 'joy', 'surprise', 'browRaise', 'upperLipRaise', 'mouthOpen', 'eyeClosure', 'cheekRaise'**

2.3.4 Questionnaire

We asked the following questions to the participants in a form each post trial.

1. Did you experience frisson while watching this video?

2. How many times did you get chills?
3. When did the frisson happen?
4. What caused the frisson to happen?
5. What are the three most meaningful ideas from the speech?
6. Were the frisson triggered by the device?
7. How intense were the frisson you experienced?
8. Did you experience cold thermal feedback from the device?
9. Did the thermal feedback caused an experience of frisson?
10. On a scale from 1 to 10, how intense was your emotion during the film?
11. Were you moved by the video?
12. How confident are you that you understood this speech?
13. Have you seen this video before?
14. If so, please compare this video watching experience to the previous one.
15. What emotions did you experience when watching the video?
16. Would you say that these emotions came from the inside or from the outside of the body?
17. Would you say that these emotions were more in the body or in the mind?
18. Do you share the speaker's feelings?
19. Do you share the speaker's viewpoint?
20. Which of these sentences do you remember best from the speech?
21. How pleasurable was this experience?
22. How relaxing was this experience?
23. How did you like the video?
24. Would you like to go through the experience again?
25. Please write a short summary of this speech in one sentence.

3. Code

R Code for testing data

```
library(psych)
library(nnet)
library(foreign)
```

```
library(Hmisc)
library(MASS)
library(extrafont)
library(PMCMR)
library(dplyr)
library(ggpubr)
library(coin)

wDev = read.csv("wDev.csv",sep = "," , stringsAsFactors = FALSE)
woDev = read.csv("woDev.csv",sep = "," , stringsAsFactors = FALSE)


wDev[11,4] = 10
woDev[4,4] = 1
woDev[11,4] = 10
woDev[14,4] = 3
woDev[17,4] = 4
woDev[19,4] = 4

woDev[2,25] = 8
woDev[16,24] = 8

wDev[12,22] = 8
wDev[12,23] =  9

wDev = wDev[-c(14),]
woDev = woDev[-c(14),]

#No of Chills
 x = c(wDev[,4])
 y = c(as.numeric(woDev[,4]))
 t.test(x, y, paired = TRUE, alternative="greater")
```

```
 shapiro.test(x-y)

#Intensity
 x = c(wDev$How.intense.were.the.frisson.you.experienced.)
 y = c(woDev$How.intense.were.the.frisson.you.experienced.)
 wilcox.test(x,y,paired = TRUE, alternative="greater")
 shapiro.test(x-y)

#Emotional Intensity
 x = c(wDev$On.a.scale.from.1.to.10..how.intense.was.your.emotion.during.the.film.)
 y = c(woDev$On.a.scale.from.1.to.10..how.intense.was.your.emotion.during.the.film.)
 wilcox.test(x,y,paired = TRUE, alternative="greater")
 shapiro.test(x-y)

#Moved by the Video
 x = c(wDev$Were.you.moved.by.the.video.)
 y = c(woDev$Were.you.moved.by.the.video.)
 wilcox.test(x,y,paired = TRUE, alternative="greater")
 shapiro.test(x-y)

 #Confident
 x = c(wDev$How.confident.are.you.that.you.understood.this.speech.)
 y = c(woDev$How.confident.are.you.that.you.understood.this.speech.)
 wilcox.test(x,y,paired = TRUE, alternative="two.sided")
 shapiro.test(x-y)

 #Speaker Feeling
 x = c(wDev$Do.you.share.the.speaker.s.feelings.)
 y = c(woDev$Do.you.share.the.speaker.s.feelings.)
 t.test(x, y, paired = TRUE, alternative="greater")
 wilcox.test(x,y,paired = TRUE, alternative="greater")
 shapiro.test(x-y)
```

```
#Speaker Viewpoint
x = c(wDev$Do.you.share.the.speaker.s.viewpoint.)
y = c(woDev$Do.you.share.the.speaker.s.viewpoint.)
wilcox.test(x,y,paired = TRUE, alternative="greater")
shapiro.test(x-y)

#Body and Mind
x = c(wDev$Would.you.say.that.these.emotions.were.more.in.the.body.or.in.the.mind.)
y = c(woDev$Would.you.say.that.these.emotions.were.more.in.the.body.or.in.the.mind.)
wilcox.test(x,y,paired = TRUE, alternative="both")
shapiro.test(x-y)


#Inside and Outside
x = c(wDev$Would.you.say.that.these.emotions.came.from.the.inside.or.from.the.outside.of.the.body.)
y = c(woDev$Would.you.say.that.these.emotions.came.from.the.inside.or.from.the.outside.of.the.body.)
wilcox.test(x,y,paired = TRUE, alternative="two.sided")
shapiro.test(x-y)



#Pleasurable
x = c(wDev$How.pleasurable.was.this.experience.)
y = c(woDev$How.pleasurable.was.this.experience.)
wilcox.test(x,y,paired = TRUE, alternative="greater")
shapiro.test(x-y)
```

```
#Relax
x = c(wDev$How.relaxing.was.this.experience.)
y = c(woDev$How.relaxing.was.this.experience.)
wilcox.test(x,y,paired = TRUE, alternative="greater")
shapiro.test(x-y)

#Like the video
x = c(wDev$How.did.you.like.the.video.)
y = c(woDev$How.did.you.like.the.video.)
wilcox.test(x,y,paired = TRUE, alternative="greater")
shapiro.test(x-y)



values = c(x,y)
labl = "Pleasure"
name = c(rep("wDev",21),rep("woDev",21))
c1 <- rainbow(2)
c2 <- rainbow(2, alpha=0.2)
c3 <- rainbow(2, v=0.7)
boxplot(values~name, ylab =
labl,cex.lab=1.5,cex.axis=1.5,col=c2,par(mai=c(.5,1.0,0.82,0.42)),boxwex = 0.4, medcol=c3,
whiskcol=c1, staplecol=c3, boxcol=c3, outcol=c3, pch=23)
```

3. **Data Availability Statement**

All supporting data is available at public repository: https://doi.org/10.7910/DVN/E4ZYOT
